# The complexity of leadership in coproduction practices: a guiding framework based on a systematic literature review

**DOI:** 10.1186/s12913-024-10549-4

**Published:** 2024-02-17

**Authors:** Sofia Kjellström, Sophie Sarre, Daniel Masterson

**Affiliations:** 1 The Jönköping Academy for Improvement of Health and Welfare, School of Health and Welfare, Jönköping University, Barnarpsgatan 39, Jönköping, Sweden; 2 Florence Nightingale Faculty of Nursing, Midwifery & Palliative Care, King’s College London, London, UK

**Keywords:** Management, Leadership, Coproduction, Health and welfare

## Abstract

**Background:**

As coproduction in public services increases, understanding the role of leadership in this context is essential to the tasks of establishing relational partnerships and addressing power differentials among groups. The aims of this review are to explore models of coproduction leadership and the processes involved in leading coproduction as well as, based on that exploration, to develop a guiding framework for coproduction practices.

**Methods:**

A systematic review that synthesizes the evidence reported by 73 papers related to coproduction of health and welfare.

**Results:**

Despite the fact that models of coleadership and collective leadership exhibit a better fit with the relational character of coproduction, the majority of the articles included in this review employed a leader-centric underlying theory. The practice of coproduction leadership is a complex activity pertaining to interactions among people, encompassing nine essential practices: initiating, power-sharing, training, supporting, establishing trust, communicating, networking, orchestration, and implementation.

**Conclusions:**

This paper proposes a novel framework for coproduction leadership practices based on a systematic review of the literature and a set of reflective questions. This framework aims to help coproduction leaders and participants understand the complexity, diversity, and flexibility of coproduction leadership and to challenge and enhance their capacity to collaborate effectively.

**Supplementary Information:**

The online version contains supplementary material available at 10.1186/s12913-024-10549-4.

## Introduction

For more than 40 years, scholars and practitioners have sought to identify and understand various aspects of coproduction with the goal of improving services as well as equalizing (or at least reorganizing) power relations in service design and delivery [1]. More recently, such discussion has focused on the roles of leaders and leadership in coproduction, seeking to describe and assess the various types of leaders and leadership that might maximize the goals of coproduction processes and outcomes. Leaders can act to make coproduction, in all its forms, *happen* [2, 3]. Leaders can enhance coproduction by providing resources, establishing inviting structures, and prioritizing the involvement of various stakeholders. Conversely, they can inhibit coproduction by perpetuating conservative administrative cultures, failing to provide training, or being reluctant to share power [3]. Coproduction relies on leadership at all levels, ranging from senior managers to local “champions” and including the citizens and third-sector organizations that participate in coproduction activities and practices.

This review presents a synthesis of research on the leadership of coproduction, which has been recognized for its scarcity [3–6]. The review provides new knowledge regarding the fact that coproduction leadership must become more deliberately (in)formed by collective leadership models. It also illustrates the multiplicity and complexity associated with coproduction leadership activities by outlining practices in which leaders must engage to ensure success. This review can inform a framework that offers guiding insights on which commissioners, evaluators, managers and leaders of coproduction can reflect as well as suggestions and directions for future research.

### Coproduction

Coproduction is a broad concept that is associated with different meanings across a range of contexts [1]. Many definitions and uses of the term coproduction and codesign have been identified [7]. Throughout this paper, although we acknowledge the distinctions associated with the concepts and origins of the notion of codesign, we use the broad term coproduction to refer to some form of collaboration or partnership between service providers and service users or citizens. For this review, we follow the definitions provided by Osborne and Strokosch [8], who identified ‘*consumer coproduction’* as an inevitable component of value creation in interactions among service providers; *‘participatory coproduction’,* in which context participation is deliberative and occurs at the strategic level of service design and planning; and *‘enhanced coproduction’,* which represents a potential mechanism for transforming organizational processes and boundaries.

Power is inevitably central to coproduction. Schlappa and Ymani claimed that the coproduction process is “inherently negotiated, emergent and reliant on a range of actors who may have both common and contrasting motivations, and are able to exercise power, which in turn is moderated by the context in which these relations occur” [6]. This sensitivity to motivation, context and power is helpful for our understanding of leadership in coproduction.

### Leadership models

Most conceptualizations of leadership have been based on the claim that leadership is a kind of inherent characteristic exhibited by human beings, such that leaders are depicted as heroes with unique traits, styles or behaviours [9]. However, research on leadership in coproduction is important in relation to an emerging body of research that focuses on the notion of “leadership in the plural” [10] or “collective leadership” [11, 12]. These phrases act as umbrella terms that refer to overlapping concepts such as shared, collaborative, distributed, pooled and relational leadership. A core feature of these models is that leadership is not (only) viewed as a property of individuals and their behaviours but rather as a collective phenomenon that is distributed or shared among different people [10]. A distinction can be made between two types of collective leadership. Leadership can be shared in interpersonal relationships; for example, it can be pooled among duos or trios at the top of an organization, or shared leadership can be exercised within teams working on a project. This notion is based upon the assumption that people have different skills that complement each other. The second kind of collective leadership is a more radical version of this notion, according to which leadership emerges as a result of direction, alignment, and commitment within a group [11] or can be observed to reside within the system, for example, in the form of distributed leadership across interorganizational and intraorganizational boundaries and networks [10, 12]. In cross-sectoral collaboration, leadership is distributed across time and space, which requires structures to guide how leadership is shared and organized. It has been argued that collective leadership is best suited to the analysis of coproduction practices [4, 6, 13, 14].

It is important to note that distinctions have been made between management (planning, monitoring and controlling) and leadership (creating a vision, inspiring and changing) based on behaviours [15]. However, many authors have not made such a distinction, and the terms have frequently been used interchangeably. We therefore adopt the practice employed in the papers included in this review and use the terms leadership and leader as catch-all terms; we only use the words management or manager when the papers refer to job titles or ‘public management’.

Leadership models can be regarded as resembling a colour palette that offers a variety of choices, and similar to colours, some models fit a situation better than others. This paper investigates the use and fit of various leadership models for coproduction.

### Leadership of coproduction research

Extant research on the leadership of coproduction has been described as “sparse” [4], a “neglected area” [5] and “overlooked” [3, 6]. Despite a recent resurgence of interest in the potential of coproduction as a means of maintaining and improving the quality of health and social care, significant questions regarding how coproduction can and should be led in this context remain unanswered. Most reviews of coproduction have not addressed this issue [2, 16–18]. Clarke et al.’s (2017) review identified the lack of managerial authority and leadership as a key barrier to the implementation of coproduced interventions but did not explore the implications of this finding for future practice. The review conducted by Bussu and Galanti (2018) stands alone in its focus on leadership, although the empirical cases explored by those authors were restricted to the context of local government in the UK. Recent empirical case studies that have explored leadership [13–15, 19] have focused on public managers [3, 5, 14] or on identifying the consequences of different models of leadership. This review contributes to the literature by providing knowledge regarding how to make deliberate choices pertaining to coproduction leadership in terms of how it is conceptualized and shared and the activities that are necessary for leading coproduction.

### Coproduction leadership practices

The leadership of coproduction poses a number of challenges. A proposed aim of coproduction is to drive change within services and in traditional state-citizen relationships by establishing equal and reciprocal relationships among professionals, the people using services, and their families and neighbours. This task requires a restructuring of health and welfare services to equalize power between providers and other stakeholders with an interest in the design and provision of these services. However, it has been suggested that coproduction runs the risk of reproducing existing inequalities in power rather than mitigating them since coproduction is inevitably saturated with unequal power relations that must be acknowledged but cannot be managed away [20].

### Aim

In this paper, we present the findings of a systematic review of the literature on leadership in coproduction. The purpose of this review is to explore models of coproduction leadership and the practices involved in leading coproduction in the context of health and social care sectors [7]. The results are synthesized to develop a framework for actors who seek to commission, design, lead or evaluate coproduction processes. This framework emphasizes the need to make more deliberate choices regarding the underlying conceptualization of leadership and the ways in which such a conceptualization is related to the activities necessary for leading coproduction. Based on the framework, we also propose specific guiding questions for individuals involved in coproduction in practice and make suggestions for future research.

## Method

**Fig. 1:**
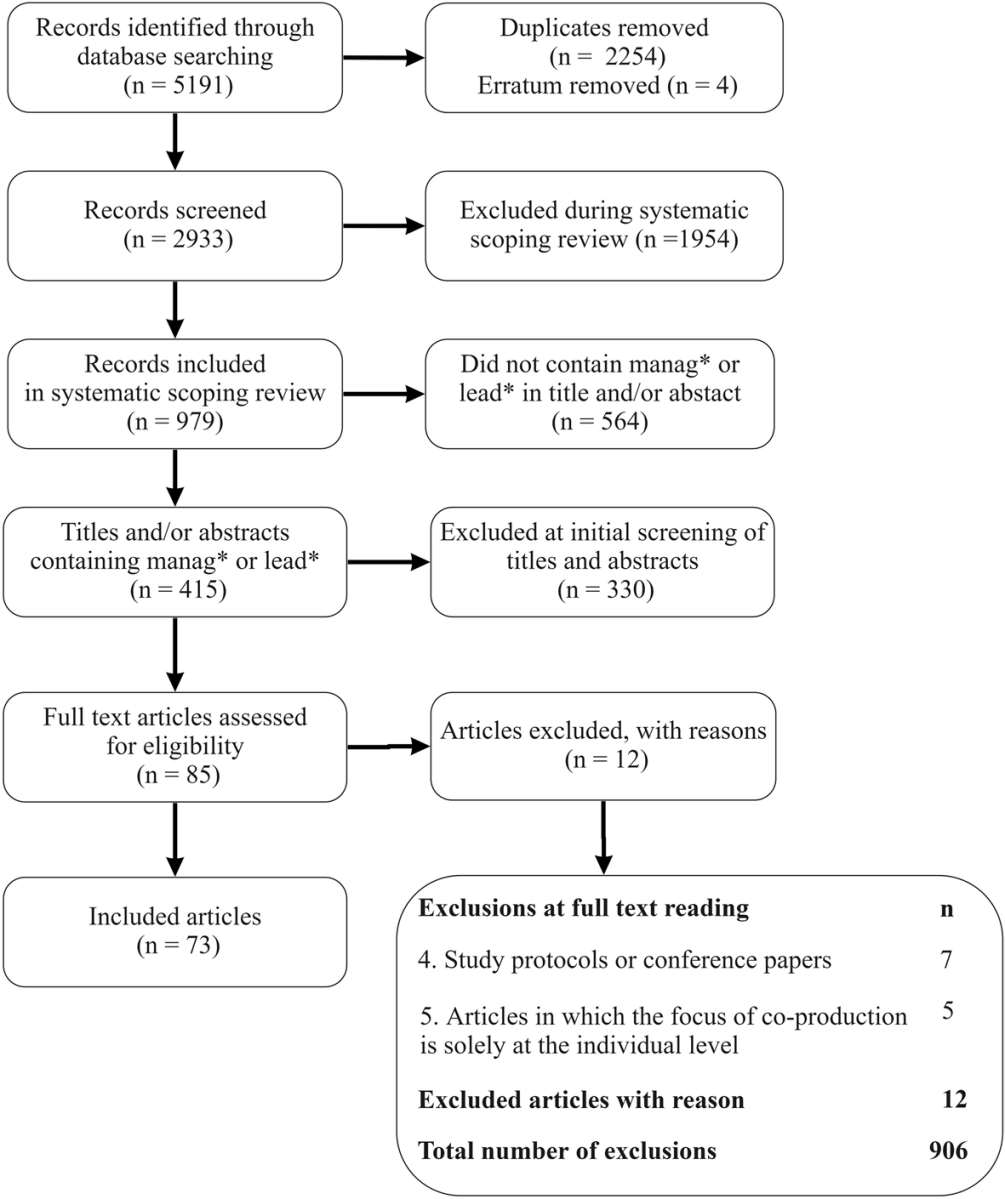
PRISMA flow chart

This systematic literature review is based on a study protocol on coproduction research in the context of health and social care sectors [21], and data were obtained from a published scoping review, where the full search strategy is provided [7]. The scoping review set out to identify ‘what is out there’ and to explore the definitions of the concepts of coproduction and codesign. In brief, the following search terms for the relevant concept (co-produc* OR coproduc* OR co-design* OR codesign*) and context (health OR social OR & “public service*” OR “public sector”) were used to query the following databases: CINAHL with Full Text (EBSCOHost), Cochrane Central Register of Controlled Trials (Wiley), MEDLINE (EBSCOhost), PsycINFO (ProQuest), PubMed (legacy), and Scopus (Elsevier). This paper focused on leadership. All titles and abstracts included in the scoping review (*n* = 979) were obtained and searched for leadership concepts (leader* OR manage*) (*n* = 415). These materials were reviewed independently by SK and SS using the following inclusion criterion: conceptual, empirical and reflection papers that included references to the management and/or leadership of coproduction. Study protocols were excluded because we wanted to capture lessons drawn from implementation, and conference papers were excluded because they lacked sufficient detail. Articles focusing on the context of individual-level coproduction (i.e., cases in which an individual client or patient was the focus of coproduction) were excluded, as we were interested in the leadership processes involved in collective coproduction. Conflicts were resolved through discussion and further consideration of disputed papers. This process led to the inclusion of 73 articles (Fig. 1 – PRISMA flow chart).

### Analysis

The method used for this research was a systematic review with qualitative synthesis. The strength of this approach lies in its ability to complement research evidence with user and practitioner considerations [22]. In the process of examining the full texts of the papers, two researchers (SK and SS) extracted background data independently. To promote coproduction, four stakeholders were strategically selected through the personal networks of one of the authors, SK. These stakeholders exhibited diverse expertise in the leadership of coproduction. One was a leadership developer and family member of an individual with 24/7 care needs. Another was a physician. The third worked in peer support and had personal experience with mental health services. The fourth was a health care leader. Four key articles were chosen due to the diversity of leadership ideas they exhibit and the depth of the explicit text on leadership they provided. During the analysis by stakeholders, no themes were changed or refined; instead, the analysis confirmed the relevance of the initially identified themes, thus emphasizing the robustness of our findings based on a process that involved reading four key articles and identifying the perceived key implications for our research aim.

A qualitative synthesis unites the findings of individual studies in a different arrangement, thereby constructing new knowledge that is not apparent from the individual studies in isolation [23]. This fact is particularly evident in this review, since leadership was seldom the main focus of the included articles. Accordingly, we employed multiple pieces of information to construct a pattern. The process of synthesis started at a very broad level with the goal of understanding which aspects of leadership were addressed in the literature. This process then separated into two strands. One such strand focused on interpreting the data from the perspective of current leadership models, while the other focused on interpreting leadership practices – i.e., the activities and relationships that are part of the process of leading coproduction. We searched for themes both within and across individual articles, and our goal was interpretative rather than purely aggregative. This process resulted in three themes pertaining to coproduction leadership models and nine coproduction leadership practices. We present these findings together in the form of a framework because consideration of both leadership models and practices prompts better and more conscious choices, which can improve the quality of coproduction. Persons one and two from the stakeholder group also provided feedback on a draft of this paper, and their insights were integrated into this research.

### Sample description

We included 73 papers (Additional file 1) dating from 1994 to 2019 (the year in which the initial search was performed). Most of these papers were empirical (*n* = 54), and more than half of them were case studies (*n* = 30). Fifteen articles were conceptual papers, and four were literature reviews. The setting or focus of the papers was predominantly on services (*n* = 66), while the remainder of the papers were on research (*n* = 4) or policy (*n* = 3). The papers drew on evidence collected from 13 countries, and the most common national setting was the UK (*n* = 29). Nine cross-national papers were also included. Issues related to leadership were rarely the focus of the papers.

## Results: A coproduction leadership framework

The synthesis consists of three parts (roles, models and practices), which are combined to develop an overarching and integrative framework for essential issues pertaining to coproduction leadership [4, 24].

### People and roles

The way in which the leadership of coproduction has been conceptualized in the literature suggests that a range of actors are involved in the coproduction of health and wellbeing and that these actors can take on different leadership roles and functions. Service users, community members and community representatives can play a vital role in the task of deliberatively coproducing or even transforming services, as can third-sector organizations, external experts, politicians, mid-level facilitators, managers, and senior leaders.

It has been argued that it is important to involve leaders from diverse backgrounds who have personal experiential knowledge of public involvement to encourage involvement from a broader population [25–27]. Service users and community members play leadership roles in coproduction initiatives related to health or well-being. These roles involve shared decision-making and accountability at various levels, ranging from the personal to the systemic.

Senior leaders include formal representatives of organizations (executives, politicians, or formal managers) and formal or respected leaders of communities. They play an important role throughout this process. During the initiation stage, by implementing and sustaining the outcomes of coproduction, they play a crucial role in the provision of resources such as time, money, materials, and access to networks. In the interim stages, their commitment to coproduction, sponsorship, and engagement is vital.

Champions and ambassadors use their expertise and passion to drive coproduction efforts. In particular, "insider" champions can establish trust among participants and help service providers understand the importance of coproduction. These champions advocate for coproduction and actively support initiatives [28–31]. Ambassadors are individuals who have expertise and volunteer their time to train others or work with clients in coproduced services. They play a crucial role in the tasks of supporting and promoting coproduction [28, 32, 33].

Project leaders and facilitators are individuals who are responsible for guiding and supporting coproduction projects, thereby ensuring their smooth operation and collaborative nature. Project leaders are responsible for overall project management, including the setting of goals, objectives, and timelines. They play a pivotal role in ensuring that projects remain on track, and they facilitate accessible and transparent dialogue among stakeholders and ensure equal representation [34, 35]. Facilitators focus on supporting the group involved in coproduction, maintaining respectful interactions, empowering service users and carers, and addressing any tensions that may arise during the collaborative process [36, 37].

In summary, senior leaders sponsor and support coproduction. Champions and ambassadors are individuals who advocate for and support coproduction initiatives, while project leaders and facilitators are responsible for managing and guiding coproduction projects themselves, thereby ensuring effective collaboration among stakeholders. All of these roles can be played by people drawn from various backgrounds, including senior staff, health care professionals, experts in coproduction, researchers, citizens, or volunteers.

### Three models of leadership in coproduction

These actors play different leadership roles, and leadership can be exercised by individuals or groups. Three leadership models have been proposed: leadership as enacted by individual leaders, coleadership and collective leadership.

#### Leadership by individual leaders

A leader-centric view has been the dominant interpretation of leadership in the field of coproduction. Many references were made to “senior leaders”. This term was used to describe formal representatives of organizations or services (senior managers, executives), formally appointed community leaders (policy-makers, local government leaders), or respected leaders of communities. Senior support was described as an important success factor in coproduction [37–45]. Other leadership roles included project leaders, facilitators, ambassadors, and champions – as described in the previous section.

Some papers referred to traits and characteristics exhibited by leaders that facilitate coproduction. These factors included innovativeness, personability, action orientation [46], courage [47], passion [32, 46], and empathy [25, 46, 48]. “Strong leadership” was often mentioned, albeit without elaboration [49–55]. By implication, “strong leadership” appeared to include providing clear direction and guidance, having a clear vision [53], holding onto a vision [34], and keeping the vision alive for the team [43].

Other researchers noted a more collaborative and democratic leadership style that is characterized by listening, transparency, deliberation, and nurturing coproductive behaviours [27, 30, 48]. Senior leaders could use a “top-down” approach to promote user involvement. Alternatively, they could “learn to manage horizontally not top down; embrace ground up initiatives; [and] aim to empower partners” [32, 45, 51] and be “open to changes that would disturb traditional relationships and power disparities between service users and providers” [41]. Respondents to a survey of participants in a peer-led support network favoured a traditional directive model of leadership alongside a more facilitative and enabling style [56]. However, they found it challenging to transition to a more distributed and collective leadership approach.

#### Co-leadership

The terms “co-lead”, “co-leadership” and “dual leadership” refer to situations in which a formal leadership role is allocated to more than one person, in which context the relevant people may represent different institutions or different groups, e.g., different professional groups, researchers and service users/citizens, or teachers and students [28, 31, 40, 41, 57, 58]. Coleads were defined as “individuals who led and made joint decisions” [59]. Some papers explored the leadership role of service users or community members in the coproduction of *research* related to health or wellbeing [35, 60, 61]. In these studies, areas of research were proposed by patients/community members, who then collaborated with academic researchers, thereby playing an equal or leading role. Coleadership was reported to result in shared learning.

#### Collective leadership

Few discernible differences among **“**shared”, “distributed” and “collective” leadership were found in the papers included in this review. The approaches examined in this context were characterized by distributed roles and responsibilities in which different individuals’ skills and expertise were identified as best suited to the task at hand. Shared leadership depends on willingness on the part of leaders (implicitly non-community leaders) to be challenged and directed by community members rather than rigidly maintaining their previous conceptions of the issues and the appropriate means of addressing them [36].

Ward, De Brún, Beirne, Conway, Cunningham, English, Fitzsimons, Furlong, Kane and Kelly [62] referred to collective leadership as an emergent and dynamic team phenomenon. Other authors argued for a more structured approach to shared leadership [36, 41] or distributed leadership [28, 42, 56, 59, 63]. Such an approach could involve allocating specific roles to service users, engaging them in a formal structure and/or enabling them to set an agenda [41], specifying shared roles and responsibilities [36], and/or providing dedicated support to lay “champions” in research studies [28]. Various benefits were attributed to collective leadership, such as empowering people to speak up [36, 51] and feel engaged.

### Nine practices associated with leading coproduction

We identified nine processes that encompass wide-ranging activities and interactions between individuals and groups with regard to leading the coproduction of health and wellbeing. As Farr noted, “Coproduction and codesign […] involves facilitating, managing and co-ordinating a complex set of psychological, social, cultural and institutional interactions” [64]. In some cases, these processes naturally align with certain actors—for instance, senior leaders play key roles in the tasks of initiating coproduction and implementing and sustaining its results—but other processes (championing coproduction, establishing trusting relationships, and ensuring good communication) are applicable to any and all participants in the coproduction process. Similarly, some of these practices occur at particular timepoints in a coproduction arc (namely, during the stages of initiation or implementation), while others can occur at any or all timepoints (i.e., during the assimilation stage or beyond). Deliberately considering the most suitable leadership model with regard to the aims and context of an initiative is useful at the start, but reflecting on the operation and appropriateness of the model is always salient.

#### Initiating coproduction

The initiation of coproduction entails recognizing the need for coproduction, dedicating resources, inviting and establishing relevant multi-stakeholder coproduction networks, and coproducing a vision and goals.

It has been argued that senior leaders act as gatekeepers for coproduction because they must recognize the need for it [45]. Senior leaders play a role in the task of determining the extent to which communities are given the opportunity to influence service design and integration [38, 51]. Coproduction requires resources (principally time and money but also networks), which can be used to take advantage of other resources such as skills [29, 31, 34, 40]. Senior leaders often control or provide access to such resources, which means that they are best positioned to initiate coproduction initiatives [41, 65]. However, the findings of a cross-national study on the coproduction of policy showed that, in practice, senior leaders’ control over resources meant that they tended to define the means, methods and forms of participation [65].

In the task of establishing a conducive environment for coproduction, it is important to pay attention to which actors (organizations or individuals) are participating in the process [33, 42, 64, 66] and to factors that may delimit those participants or their involvement [36, 42, 67]. Several papers emphasized the need to ensure that all stakeholders are involved from the outset [37, 38, 41, 48, 51]. In the initiation stages, a shared vision should be created [36, 61, 68], goals should be coproduced, and responsibilities should be clearly allocated [65]. Role clarity, ability, and motivation have been identified as determinants of coproductive behaviour, and leaders must implement arrangements to achieve these goals for coproducers [69].

#### Power sharing

It has been argued that coproduction leadership must attend to issues pertaining to power redistribution [60, 61, 63, 64] and uphold the ideology of coproduction by promoting the values of democracy and transparency [30, 32, 70–74]. This process can occur at different levels.

At the macro system level, several cultural shifts have been implicated in the redistribution of power – a shift in current professional and stakeholder identities; more fluid, flattened and consensus-based ways of working; and a willingness to accommodate ‘messy’ issues [75]. The last of those issues was highlighted by Hopkins, Foster and Nikitin [29, s 192], who suggested that coproduction requires service providers to “sit more easily with the unknown, to be comfortable in not having all the answers.” Similarly, “The challenge is that to be transformative, power must be shared with health service users. To do this entails building new relationships and fostering a new culture in health-care institutions that is supportive of participatory approaches” [42, p 379].

At the meso level, several practices could be used to share power. Greenhalgh, Jackson, Shaw and Janamian [30] identified the importance of equitable decision-making practices and “evenly distributed power constellations.” This goal can be achieved, for instance, by ensuring that service users represent a majority on the project management committee or in codesign events with the goal of challenging dominant professional structures and discourses [37]. Other scholars called for clear roles and responsibilities [38, 59, 65]. Mulvale, Moll, Miatello, Robert, Larkin, Palmer, Powell, Gable and Girling [36] recommended the establishment of shared roles and responsibilities, the creation of a representative expert panel to resolve stalemates, and possibly the implementation of formal agreements regarding data and reporting. Importantly, however, Greenhalgh, Jackson, Shaw and Janamian [30] noted that governance structures and processes alone do not automatically overcome the subtle and inconspicuous uses of power. Farr [64] recommended the constant practice of critical reflection and dialogue and posed several questions for participants to consider: who is involved, what the interactions are like, how coproduction efforts are implemented within and across structures, and what changes are made.

Although sharing power has been described as an essential component in coproduction, the involvement of stakeholders does not necessarily entail empowerment [47], and case studies have demonstrated that service improvement initiatives that *involve* citizens or service users can be instrumental and effective with regard to improving services *without* enhancing or sharing power or political consciousness if stakeholders are invited but power is not shared [32]. Farr [64] noted that rather than coproduction being *inherently* emancipatory, coproduction and codesign processes can have either dominating or emancipatory effects [33], and the exclusion of vulnerable groups from coproduction has the potential to reinforce existing inequities [75].

#### Training and development for emerging leadership

The importance of appropriate training and mutual learning was noted in several papers [36, 42, 48, 63, 69, 76, 77]. Implicitly, training for professionals was framed in terms of training in the process of sharing power with service users or facilitating collaboration, whereas training for service users was framed as capacity-building in terms of collaboration and/or leadership. In one case study focusing on coproduced research, participants rejected the notion of “training” from academic researchers with the aim of avoiding suggesting that a certain level of “expertise” needed to be transferred [60].

Playing a leadership role can be empowering [51, 71], but for some individuals, it can be overwhelming [71]. Leading coproduction requires practice and the development of skills and capacities [26, 48]. In some initiatives, lay partners were initially involved in limited roles and gradually took on more responsible leadership tasks over time [28, 42, 78]. In addition, community members’ level of involvement was flexible—they could be participants or take on additional roles as volunteers, paid staff members or directors of organizations. This flexibility offered participants the opportunity to "begin sharing, as opposed to shouldering, the burden of involvement” [71].

#### The provision of support

Support is necessary throughout the coproduction process from its outset to the stages of implementation and sustainment [25, 34, 68]. Key dimensions of support include facilitating, advocating for, and championing coproduction. Project management is instrumental to the smooth operation and facilitation of coproduction [34, 35, 37, 44]. Several facilitation activities are conducted by project leaders and facilitators [41, 42, 59, 61, 78]. These activities include holding onto a vision and keeping it alive for the team, ensuring that the project remains on track, and helping maintain momentum. In one codesign case study, facilitators helped people focus on quick wins with the goal of maintaining motivation and engagement; they "needed to support movement from inaction to action, by sifting through group ideas to fix a plan" [34]. Although these authors acknowledged that this approach may have limited coproduction, they argued that such initiatives would not be sustainable if they were perceived to be “unfeasible.”

Another key function entails advocating for and championing coproduction initiatives to ensure that the process remains ongoing [25, 28–32, 37, 41, 74, 79]. Senior leaders play an important role in the task of championing coproduction, and their support has often been described as an important success factor [34, 38, 39, 43, 80]. However, effective champions could equally include health care professionals [37], experts in coproduction [51], researchers [35, 60, 61], volunteers [51] or other citizens [41, 61]. Champions with lived experience can gain the confidence of their peers and help create understanding among service providers [28, 36].

#### Establishing trusting relationships

Coproduction is essentially relational and requires concerted efforts to establish trusting relationships and a sense of commitment. The importance of trust among stakeholders in coproduction has been noted in several papers [28, 30, 36–38, 46, 48, 64, 74, 81, 82]. In the field of health research, it is difficult to secure funding for the process of establishing relationships and working in the context of partnerships during the early stages of development [25]. It can therefore be helpful to base recruitment for coproduction initiatives on pre-existing trusting relationships [36]. If such pre-existing trusting relationships do not exist, policy-makers and senior leaders play a role in the creation of frameworks that can facilitate the development of trust both among organizations and between organizations and citizens, such as political and bureaucratic commitment on the part of regional and local governments and the engagement of actors who play a “boundary-spanning” role in the relationships between service providers, non-government organizations and communities [38]. Trust is established based on clear responsibilities [38] and adherence to the principles of engagement in coproduction. In addition to these frameworks, individual leaders must develop trust through interactions with coproducers, using collaborative skills such as those pertaining to communication and listening [48]. In one case study, through the frank sharing of the organizational, financial, and governance challenges and opportunities faced by stakeholders, people reached a growing understanding and appreciation of each other’s positions, which engendered trust [30]. Mulvale, Moll, Miatello, Robert, Larkin, Palmer, Powell, Gable [36] highlighted the importance of understanding and responding to participants’ histories, contexts, and cultural differences.

Commitment can be viewed as more important than resources [59]. The commitment to and engagement in coproduction exhibited by an organization’s senior leaders demonstrate organizational commitment and lend credibility to coproduction initiatives [25, 34, 38, 41, 47, 59, 80, 83]. On some occasions, coproduction initiatives are reported to senior leaders, while on other occasions, the senior leaders were part of the coproduction team. Senior leaders who adopt a more hands-on approach serve as role models [25], advocating for patient engagement and engendering commitment on the part of staff and patients [28]. In public health initiatives, buy-in from community leaders confers legitimacy on innovations, helps ensure community trust [61, 78], increases the engagement of community members [78] and is key to a project’s success [83].

#### Communication

Communication is a key activity in coproduction, and leaders must establish an environment that is conducive to “epistemological tolerance” [47], such that different perspectives are valued and appreciated. Such environments facilitate dialogue among partners [28, 30, 35, 51] and allow critical voices to be heard [42]*.* Open dialogue among stakeholders is a starting point for the task of identifying the sources of assumptions and stereotypes, which is itself a prerequisite for change in attitudes and practice [28]. Project leaders must also facilitate accessible and transparent dialogue and ensure the equal representation of all stakeholders, including those who are less able to communicate verbally [57, 71]. Professional leaders are responsible for critically reviewing their professional norms, organizational/institutional processes and past and present policies and practices [55, 75].

Dealing with multiple stakeholders, which is inevitably required in coproduction, requires addressing multiple perspectives in an attempt to bring them together. This task frequently involves a degree of conflict and peace negotiation [30, 34, 41, 48, 61, 64]. Leaders should be alert to conflict and power dynamics [34, 36]. It may be necessary for meeting chairs to encourage participants to move on from their familiar, entrenched positions to avoid descending into circular arguments and stalemates (Chisholm et al. 2018). This task could require the injection of a critical voice, as Greenhalgh explained:

*“Meeting chairs were selected for their leadership qualities, ability to identify and rise above “groupthink” (bland consensus was explicitly discouraged), and commitment to ensuring that potential challenges to new ideas were identified and vigorously discussed. They set an important ethos of constructive criticism and creative innovation, with the patient experience as the central focus. They recognized that if properly handled, conflict was not merely healthy and constructive, but an essential process in achieving successful change in a complex adaptive system.*” [30]

Leaders must acknowledge the facts that discomfort can arise when more equitable relationships are established [61] and that challenges to professional identity [84] and the loss of control [72] are factors in this process.

#### Networking

Networking refers to the practice of establishing and maintaining relationships with various stakeholders both within and outside the coproduction initiative. Since coproduction involves working with different stakeholders in networks, several papers have discussed the vital mediating processes associated with this context.

*“Bridging, brokering and boundary spanning roles have a key role in cross fertilization of ideas between groups, for generating new ideas and for increasing understanding and cooperation”* [32, 53].

In policy-making, it is helpful to develop coordination structures and processes such as cross-sector working groups and committees, intersector communication channels [65], and relationship and dialogue structures [42]. Community representatives can play a mediating role between individuals and public organizations and may alleviate professionals’ concerns regarding the transaction costs of coproduction in the planning and management of services [26, 81]. However, these representatives may or may not use this power to amplify the voices of individual coproducers [81].

An important role of project leaders is that of the “broker” [32, 85], who focuses on mediating among different stakeholders in an attempt to align their perspectives [26, 37, 72, 86]. Another role focuses on spanning the boundaries across sites [50], between local service providers [68], or among local services, non-government organizations and the community [38]. Bovaird, drawing on a number of cases of coproduction, came to the following conclusion:

“*there is a need for a new type of public service professional: the coproduction development officer, who can help to overcome the reluctance of many professionals to share power with users and their communities and who can act internally in organizations (and partnerships) to broker new roles for coproduction between traditional service professionals, service managers, and the political decision-makers who shape the strategic direction of the service system.”* [81]

#### Orchestration

This practice involves reflecting on and improving coproduction itself. It includes activities such as evaluating the effectiveness of coproduction efforts, assessing the impact of coproduction on outcomes, and making adjustments to improve the coproduction process. Several papers have addressed the roles of local government or public managers or health professionals in overseeing and (as we refer to this process) ‘orchestrating’ the networks involved in coproduction at the community or local government level [30, 33, 65, 74, 87]. Orchestration involves recruiting the appropriate actors as noted above as well as directing and coordinating activities, thereby ensuring that the whole is more than the sum of its parts. As part of their orchestration work, leaders play a role in the task of managing risk in service innovation [55, 87] and must commit to self-reflexivity and a critical review of norms, policies and practices to alert themselves to any unintended negative consequences and strive to counteract them [55]. Sturmberg, Martin and O’Halloran [88] used the metaphor of ‘conducting’ to describe the function of leadership in health care – i.e., leading the orchestra through inspiration and empowerment rather than control, leading to the provision of feedback as the performance unfolds.

From a public service perspective, Powers and Thompson [69] argued that coproduction requires the leader (“usually a public official”) to mobilize the community on behalf of the public good, organize the provision of the good, create incentives, and supervise the enforcement of community norms. Sancino [74] argued that local governments play a ‘meta-coproduction role’ that requires them to maximize the coproduction and peer-production of community outcomes by taking into account community contributions and deciding which services should be commissioned or decommissioned (a point that was also made by Wilson [87]) and to promote coproduction and peer-production in such a way as to promote the coproduction of outcomes that have been decided through a democratic process. In this way, he argued,

*"the local government becomes the pivot of different kinds of relationships and networks made up of different actors who collectively assume the responsibility for implementing an overall strategic plan of the community beyond their specific roles and interests." *[74]

Sancino [74] attempted to draw out the leadership implications of this situation, arguing that rather than focusing on service delivery, public managers must create appropriate conditions for such meta-coproduction. This task entails a directing role based on framing shared scenarios for change in the community through sense-making; an activator role based on activating, mobilizing and consolidating the social capital of the community to promote diffused public leadership; a convenor role based on serving as a meta-manager in the process of self-organizing the knowledge, resources and competencies pertaining to the community in question; and an empowering role based on creating conditions in which peer production and coproduction can be combined to create the corresponding added value (i.e., higher levels of community outcomes) [74]. This practice essentially focuses on self-assessment and continuous improvement within the coproduction framework.

#### Implementation

It has been argued that coproduction in services [30, 79] or policy-making [65] may improve implementation. The role of leadership in supporting the implementation of the outcomes of coproduction is essential [37, 41, 49, 52, 64, 65, 85, 86]. Leaders can argue for the legitimacy of coproduced innovations [89] and implement mechanisms aimed at acting on the issues thus raised and continuing to promote patients’ involvement [28, 41]. Implementing the outcomes of coproduction relies on outcome-focused leadership [30]. The results of coproduction initiatives must be transformed into strategic plans and policies [41], and patient perspectives must be translated into actionable quality improvement initiatives [49]. Conversely, implementation can be blocked by leaders who fail to respond to the results of coproduction initiatives or who implement policies or procedures that are poorly aligned with the recommendations arising from coproduction [30, 41]. It should also be acknowledged that not all demands thus generated can always be met [61]. Failures of implementation run the risk of stakeholder disillusionment; thus, the management of expectations is important.

### A framework for coproduction leadership

When coproduction is initiated, it is possible to consider the actors involved and to imagine various forms of coproduction. In the design process, it is possible to make a deliberate choice with regard to the most appropriate model of leadership, and depending on the leadership model selected (leader-centric, coleadership, or collective leadership), different leadership practices emerge. The nine leadership practices identified can be enacted by different people and in different ways. The leadership of coproduction that thus emerges is shaped by issues such as the model of coproduction, the stakeholders involved, participants’ motivations and the context of coproduction. A main concern lies in the need to design project structures and work practices that are aligned and that enable leadership to emerge. We thus created a table (Table 1) that illustrates potential reflective questions in this context.

**Table 1:** Guiding reflective questions for coproduction leadership

*Topic*	*Guiding considerations*
***People and roles***	- Are all relevant and affected people in the room? (senior leaders, people/citizens, politicians, professionals etc.) - Do you need project leaders, facilitators, champions or ambassadors? - How are leadership roles working for everybody?
***Models of leadership***	- When and how is a leadership model decided upon, and by whom? - Individual leadership: Are the people involved committed to sharing power and leadership? - Coleadership: How and when do individuals share the leadership? - Collective leadership: How are conditions enabled so that leadership emerges within a group? How will the group’s engagement be enhanced in leadership?
***Practices of leading coproduction***	
***1.Initiating coproduction***	- How can organizations and relevant people effectively recognize the need for coproduction? - What leadership model is most appropriate for our aims and context? - Have the necessary resources been identified and secured and how can leaders manage these? - Are all relevant and affected people in the room? (senior leaders, people/citizens, politicians, professionals etc.) - Do you need project leaders, facilitators, champions or ambassadors? - How can stakeholders be actively engaged from the beginning?
***2.Power sharing***	- How can coproduction leadership effectively address power redistribution, and what cultural shifts and practices are essential to make this redistribution successful? - What distinguishes stakeholder involvement from meaningful engagement? - How can organizations ensure that coproduction efforts do not inadvertently reinforce existing inequities and disparities, especially when involving vulnerable or marginalized groups? - Are we using the most appropriate leadership model for our aims and context? - How are leadership roles working for everybody?
***3.Training and Development***	- What strategies should leaders consider to develop coleadership skills over time, without overwhelming them? - What measures can leaders put in place to support individuals transitioning into co-leadership roles?
***4.Support***	- How can leaders strike a balance between actively facilitating coproduction processes and sharing power, responsibility and ownership that drive sustainable engagement? - How can champions of coproduction be supported within diverse stakeholder groups, recognizing that effective champions can emerge from various backgrounds and roles? - How can leaders, particularly senior leaders, advocate for and champion coproduction initiatives within their organizations or institutions?
***5.Establishing trusting relationships***	- How can leaders proactively build, facilitate and nurture trust-building efforts among diverse stakeholders involved in coproduction? - What specific actions and strategies can senior leaders employ to demonstrate and instil their commitment to coproduction initiatives to staff and participants? - In what ways can community leaders play a pivotal role in building and sustaining trust within communities and between community members and external organizations?
***6.Communication***	- How can conflict and disagreement be appreciated and productively engaged with? - How can we actively create an environment of "epistemological tolerance" that encourages diverse perspectives to be valued and embraced, enabling meaningful dialogue and constructive exchanges among coproduction partners? - What strategies and approaches can leaders employ to effectively manage conflicts and power dynamics that may arise during coproduction processes, ensuring that disagreements lead to productive discussions and decisions rather than impeding progress? - How can leaders help participants navigate the discomfort that may arise when transitioning to more equitable relationships and confronting challenges to professional identities, ensuring that these difficulties do not hinder the coproduction process but rather contribute to its growth and evolution?
***7.Networking***	- How do we network and bring different organizations and stakeholders together? - How do we bridge differences? - Are we thinking broadly enough about which actors are valuable in our system? - How can project leaders effectively mediate between various stakeholders in co-production initiatives to ensure alignment of perspectives and collaborative efforts, while avoiding power imbalances or conflicts? - What strategies can be employed to bridge gaps between different sectors or organizations, to facilitate the cross-fertilization of ideas and promote cooperation in coproduction? - How can leaders maximize the benefits of boundary spanning between sites or local service providers enhance the outcomes and sustainability of coproduction initiatives?
***8.Orchestration***	- Is there a person or body overseeing the whole process? - How can leaders effectively orchestrate and coordinate diverse stakeholders in co-production efforts to ensure that the collective effort surpasses the sum of individual contributions? - How can leaders systematically evaluate the effectiveness of coproduction efforts, including assessing their impact on outcomes, to inform ongoing improvements and adjustments in the coproduction process?
***9.Implementation***	- Do senior leaders support necessary implementation and/or sustainment? - What leadership strategies and mechanisms can effectively support the implementation of coproduction outcomes, ensuring that they are integrated into strategic plans, policies, and quality improvement initiatives? - What strategies can leaders employ to address and overcome barriers to implementation, especially when there is resistance or poor alignment between coproduction recommendations and policies or procedures?

## Discussion

This discussion highlights and problematizes the two main findings of this systematic review, namely, the need to deliberately consider underlying models of leadership and the complex character of leading coproduction.

### The need for the deliberate use of leadership in the plural

A focus on leader-centric approaches and the quality of leaders has characterized public leadership research [90]. Such a focus is echoed in our findings on coproduction leadership, first with regard to the prominence of senior leaders and, to a lesser extent, facilitators. Politicians were rarely identified in the papers included in our review despite representing some of the main actors identified in a previous review [4, 91]. Second, many papers referenced the need for “strong” leaders, and the skills and behaviours of individual leaders were noted. As other researchers have found, despite the focus of this field on relationships and interactions, its emphasis has frequently remained on the individual leader and their ability to engage and inspire followers [13]. Furthermore, even in papers that emphasized ‘coleadership’ or ‘collective leadership,’ the focus remained on public managers, service managers and facilitators. Very little evidence has been reported concerning individual service users’ or citizens’ *leadership of* (as distinct from *involvement in*) coproduction. Although the involvement of community leaders was reported to play a role in project success, no articles explored this issue.

However, some important exceptions should be noted. For example, some studies exhibited a preference for mixed models, employing both a directive approach (particularly in the beginning) and a more facilitative and distributed leadership approach [56]. Rycroft-Malone, Burton, Wilkinson, Harvey, McCormack, Baker, Dopson, Graham, Staniszewska and Thompson [53] concluded that consideration should be given to models that combine hierarchical, directive structures with distributed facilitative forms of leadership.

One explanation for this rather narrow view of leadership is that despite the rapidly increasing number of publications in the general field of coproduction [7, 18], empirical studies have still lacked depth with regard to investigations of the leadership of this process. Most empirical studies included in this review mentioned leadership only in passing or derived some conclusions regarding leadership from case studies focusing on other aspects of the coproduction process.

Another explanation for this situation is that although coproduction focuses on partnership, in most cases, senior leaders have control over resources and the power to define the means, methods, extent and forms of participation [65]. Even shared leadership models seem to rely on traditional leaders’ willingness to share power [10], as leaders are the actors who invite, facilitate, and support the participation of coleaders. However, some signs of change towards a broader view should be noted. Recent publications have theorized the leadership of coproduction and included case studies that have demonstrated leadership to be a social, collective and relational phenomenon that emerges as a property of interactions among individuals in given contexts [13, 19].

### The complexity of coproduction leadership practices

Our findings indicate that the leadership of coproduction practices entails challenging and complex tasks. Complexity emerges in cases in which many parts are interrelated in multiple ways. Different kinds of leadership activities may be necessary depending on the stakeholders involved [92], the context [13], and the mode, level, and phase of coproduction [93]. A complexity perspective based on systems thinking is therefore useful [13, 19]. All actors involved in coproduction are potential leaders, but for that potential to be realized, the coproduction initiative and its leadership must be framed and comprehended in a more plural way. A recent study on systems thinking and complex adaptive thinking as means of initiating coproduction advocated a collective leadership approach [19].

Our findings highlight the need for a complex way of making meaning of leadership throughout the coproduction process, such as the ability to be flexible due to circumstances and employ both strong leadership and more facilitative approaches when necessary. Leaders must also promote the values of democracy, transparency and the redistribution of power among stakeholders throughout the process [64, 94]. These practices and tasks are complex, which must be matched by an inner mental complexity [95, 96]. Several practices identified in this research, such as genuinely valuing diverse perspectives, promoting mutual transformative power sharing and welcoming conflicts, require a complex mode of meaning-making that results from psychological development. These issues warrant further exploration. Future studies featuring a thoughtful choice of leadership and complexity models as well as a broader methodological repertoire are thus necessary (see Table 2 for an overview).

**Table 2:** Suggestions for further research

*Topic*	*Future research agenda*
*People and roles*	- Studies on people currently obscure in current studies such as politicians, community leaders, citizens, and users
*Models of leadership*	- Studies on the emergence of collective leadership - Observation studies focussing on interactions where power and leadership emerge and are negotiated in real time, to illuminate collective coproduction leadership processes - Studies on sense-making of leadership and coproduction - Research based on collective leadership theories - Studies to explore a ‘good’ balance of strong and directive leadership with facilitative and shared leadership
*Practices of leading coproduction*	- Observational studies of leadership practices, barriers and facilitators - Qualitative longitudinal studies on the evolution and sequencing of different leadership theories and practices over time - Designing and testing different practices - Case studies focussing on training and development in the leadership and management of coproduction *in the context of the system* for all peoples - Participatory research methods like action research and interactive research (based on partnerships between researchers, patient/citizens and professional service organisations in knowledge coproduction) - Theories of systems thinking, social network theory, and complex adaptive systems theory as providing new perspectives - Investigating collective leadership practices through the lens of models such as relational leadership, and the Direction, Alignment and Commitment (DAC) model [11]

### Methodological strengths and limitations

A strength of this review lies in its integration of research on the sparse and overlooked issue of leadership in coproduction. Our search strategy, which involved using the key words manag* and lead*, may have excluded some relevant papers. To verify that this approach did not represent an excessively blunt exclusion criterion, we checked 10% of the articles that were excluded based on this criterion. All of these articles would also have been excluded for failing to include any exploration of the management or leadership of coproduction. We therefore determined that this exclusion criterion was justifiable. Many papers did not have an explicit focus on leadership; however, by synthesizing the data, all data were treated as reflections that jointly created a larger pattern, similar to a kaleidoscope. The exclusion of non-peer-reviewed papers is likely to have led to the exclusion of coproduced outputs, which may have offered important insights into the leadership of coproduction, particularly with regard to the experiences of service users and citizens playing leadership roles. In the reporting of this review, the PRISMA guidelines were followed (Additional file 2). It should be noted that the lack of reporting bias assessment and certainty assessment represents a limitation of this study.

### Future research

Future research (see Table 2) should focus on under-represented roles, such as those of politicians and community leaders, and explore emerging collective leadership models based on real-time observational studies. It should also investigate the balance between strong and shared leadership by using qualitative and participatory research methods. Incorporating systems thinking and relevant leadership models can offer new perspectives on collective leadership practices.

### Practical implications

This paper explored coproduction leadership practices and revealed that they require a deliberate and plural understanding of leadership roles and tasks. We proposed a framework for coproduction leadership that takes into account the actors involved, the models of leadership, and the leadership practices that emerge in different contexts and during different phases of coproduction. We also provided a set of reflective considerations that can help all actors involved in this process make more deliberate choices regarding the parties involved, leadership models of coproduction, and practices (Table 1).

Our systematic review revealed some gaps in the literature on coproduction leadership, such as the lack of attention to the mental complexity of coproduction leaders, the under-representation of service users and citizens as leaders, and the need for more empirical studies that use appropriate models and methods to capture the complexity of coproduction leadership. We suggest that future research should address these gaps, thus contributing to the advancement of coproduction theory and practice.

Our framework also has some practical implications for coproduction leaders and participants. At the start of coproduction process, all people, particularly leaders, must learn more about different models of leadership and how power is shared. Throughout this process, flexibility is necessary because leadership constellations change over time; they emerge and fade away, thus implying different underlying leadership models. A multitude of practices must be implemented throughout the coproduction process. People in leader roles must be aware of their personal strengths and limitations, not only with the goal of sharing leadership but also with the aim of establishing partnerships with others who have competence in certain practices, such as facilitation or addressing conflicts. Reflecting upon the guiding questions can also help illustrate the extent to which power and leadership are being shared. In conclusion, to create more equal power relations over time, we must challenge our current practices and work deliberately to enhance the capacity of individuals and groups to effectively engage in coproduction leadership.

### Supplementary Information

**Additional file 1: Appendix 1. **Description of included papers. 

**Additional file 2: Appendix 2. **PRISMA_2020_checklist - Mangement review.
