# Correction: The complexity of leadership in coproduction practices: a guiding framework based on a systematic literature review

**DOI:** 10.1186/s12913-024-10811-9

**Published:** 2024-03-13

**Authors:** Sofia Kjellström, Sophie Sarre, Daniel Masterson

**Affiliations:** 1 The Jönköping Academy for Improvement of Health and Welfare, School of Health and Welfare, Jönköping University, Barnarpsgatan 39, Jönköping, Sweden; 2 Florence Nightingale Faculty of Nursing, Midwifery & Palliative Care, King’s College London, London, UK

**Correction to: BMC Health Services Research (2024) 24:219**

10.1186/s12913-024-10549-4

In this article, affiliation 2 (Florence Nightingale Faculty of Nursing, Midwifery & Palliative Care, King’s College London, London, UK) was incorrectly assigned as a second affiliation for the author Sofia Kjellström due to a typesetting mistake. The sole affiliation for Sofia Kjellström should be affiliation 1 (The Jönköping Academy for Improvement of Health and Welfare, School of Health and Welfare, Jönköping University, Barnarpsgatan 39, Jönköping, Sweden).

This error is corrected in the author list of this Correction article and the original article has been updated. The publisher apologises to the authors and readers for the inconvenience caused by this error.

