# Supplementary material for: The complexity of leadership in coproduction practices: a guiding framework based on a systematic literature review
Source: BMC Health Serv Res. 2024 Feb 17;24:219. doi: 10.1186/s12913-024-10549-4 (PMC10873973; doi:10.1186/s12913-024-10549-4)
Supplement: Supplementary file 1 — Additional file 1: Appendix 1. Description of included papers. [file 12913_2024_10549_MOESM1_ESM.docx]

**Appendix 1 Description of included papers**

| **Author** | **Year** | **Title** | **Journal** | **Study type** | **Setting - Country** | **Setting - Field** | **Setting - other** |
| --- | --- | --- | --- | --- | --- | --- | --- |
| Anderson | 2015 | Adaptive Leadership Framework for Chronic Illness Framing a Research Agenda for Transforming Care Delivery | Advances in Nursing Science | Conceptual | Not stated | Health services | Chronic conditions |
| Andrews | 2013 | Social Capital, management capacity and public Service Performance: Evidence from the US states | Public Management Review | Quantitative | US | General public services | National level data |
| Bagot, KL; et al. | 2017 | Transitioning from a single-site pilot project to a state-wide regional telehealth service: The experience from the Victorian Stroke Telemedicine programme | Journal of Telemedicine and Telecare Quarterly | Case study | Australia | Health services | Telemedicine stroke service, Victoria |
| Bak, K; et al. | 2018 | Patient and Staff Engagement in Health System Improvement: A Qualitative Evaluation of the Experience-Based Co-design Approach in Canada | Healthcare Quarterly | Mixed qualitative & quantitative methods | Canada | Health services | Healthcare, Ontario |
| Beckett | 2018 | Embracing complexity and uncertainty to create impact: exploring the processes and transformative potential of co-produced research through development of a social impact model | BMC Health Research Policy  And Systems | Case study | Canada & UK | Health research | N=6 case studies of the coproduction of research |
| Bell et al | 2019 | Co-building a patient-oriented research curriculum in Canada | Research Involvement and Engagement | Case study | Canada | Health research | Co-design of a course in patient-oriented research |
| Bombard et al | 2018 | Engaging patients to improve quality of care: a systematic review | Implementation Science | Review | Canada | Health services |  |
| Bovaird | 2006 | Developing New Forms of partnership with the market in the procurement of public services | Public Administration | Case study | UK | General public services | Procurement of public services |
| Bovaird, T. | 2007 | Beyond engagement and participation: User and community coproduction of public services. | Public Administration Review | Conceptual | UK | General public services | User and community coproduction of services |
| Bruce, G; et al. | 2011 | Connected care re-visited: Hartlepool and beyond | Journal of Integrated Care | Named coproduction method | UK | Health and social care | Voluntary organization implementing ‘Connected Care’ |
| Budge, G; et al. | 2019 | “It kind of fosters a culture of interdependence”: A participatory appraisal study exploring participants' experiences of the democratic processes of a peer-led organisation | Journal of Community and Applied Social Psychology | Case study | UK | Health services | South-west of England, peer led, mental health Community Interest Company |
| Burhouse et al | 2015 | Coaching for recovery | BMJ Quality Improvement Reports | Qualitative | UK | Health / education services | Recovery college |
| Burhouse et al | 2017 | Preventing cerebral palsy in preterm labour: a multi-organisational quality  improvement approach to the adoption  and spread of magnesium sulphate  for neuroprotection | BMJ Quality Improvement Reports | Mixed quantitative & qualitative methods | UK | Health services | Cerebral palsy, West of England |
| Byrne, L; et al. | 2018 | The global need for lived experience leadership | Psychiatric Rehabilitation Journal | Conceptual | Global | Health services | Mental health |
| Chisholm, L; et al. | 2018 | Processes in an Experience-Based Co-Design Project with Family Carers in Community Mental Health | SAGE Open | Named co-production method | UK | Health services | Community mental health (outer London) |
| Cooke, J; et al. | 2017 | Seeing the Difference: The Importance of Visibility and Action as a Mark of Authenticity in Co-production | International Journal of Health Policy Management | Conceptual | UK | Health services | Healthcare |
| Cox, N; et al. | 2016 | A safe place to reflect on the meaning of recovery: a recovery community co-productive approach using multimedia interviewing technology | Drugs and Alcohol Today | Case study | UK | Health / education services | Recovery college, England |
| Ersoy, A | 2016 | The spread of coproduction: How the concept reached the northernmost city in the UK | Local Economy | Case study | UK | General public services | Aberdeen City Council, Scotland |
| Farmer | 2017 | Citizen participation in health services co-production: a roadmap for navigating participation types and outcomes | Australian Journal of Primary Health | Conceptual | Australia | Health services | Primary healthcare |
| Farmer | 2015 | An exploration of the longer-term impacts of community participation in rural health services design | Social Science and Medicine | Qualitative | UK | Health services | Scottish rural health services |
| Farmer | 2018 | Applying social innovation theory to examine how community co-designed health services develop: using a case study approach and mixed methods | BMC Health Services Research | Case study | Australia | Health services | Health services in a rural community |
| Farooqi, SA | 2016 | Co-production: what makes co-production work? Evidence from Pakistan | International Journal of Public Sector Management | Case study | Pakistan | General public services | Two local government areas |
| Farr | 2018 | Power dynamics and collaborative mechanisms in co-production and co-design processes | Critical Social Policy | Case study | UK | Health services | Experienced-Based Co-Design in 1) hospital breast cancer and 2) a local government innovation team |
| Gallan | 2012 | Customer positivity and participation in services: an empirical test in a health care context | Journal of the Academy of Marketing Science | Quantitative | US | Health services | Co-creation service, medical clinic |
| Gillard | 2016 | Evaluation the Prosper peer-led peer support network: a participatory coproduced evaluation | Mental Health and Social Inclusion | Mixed methods | UK | Health services | ‘Prosper’ peer-led peer support network and social movement |
| Green, S; et al. | 2018 | Implementing guidelines on physical health in the acute mental health setting: a quality improvement approach | International Journal of Mental Health Systems | Case study with quantitative outcome measures | UK | Health services | n=1 mental health ward in London |
| Greenhalgh | 2016 | Achieving Research Impact Through Co-creation in Community-Based Health Services: Literature Review and Case Study | Milbank Quarterly | Review | Australia | Health research | Models of knowledge co-creation applied to a case study of a community-based, health research-service partnership |
| Greenwood | 2019 | Development of the Intercultural Diabetes Online Community Research Council: Codesign and Social Media Processes | Journal of Diabetes Science and Technology | Case study | US | Health services | Diabetes |
| Hafford-Letchfield, T; et al. | 2018 | Developing inclusive residential care for older lesbian, gay, bisexual and trans (LGBT) people: An evaluation of the Care Home Challenge action research project | Health and Social Care in the Community | Qualitative | UK | Health and social care | Residential care for older people |
| Hämäläinen | 2016 | Cross-sector cooperation in health-enhancing physical activity policy-making: more potential than achievements? | BMC Health Research Policy and Systems | Case study | Europe | Policy | European policy |
| Haynes, E; et al. | 2019 | Community-based participatory action research on rheumatic heart disease in an Australian Aboriginal homeland: Evaluation of the 'On track watch' project | Evaluation and Program Planning | Mixed methods | Australia | Health services & health research | Heart disease in Aboriginal people |
| Hogan, MJ; et al. | 2015 | Consulting with citizens in the design of wellbeing measures and policies: Lessons from a systems science application | Social Indicators Research | Conceptual | Cross national | Policy | National and international wellbeing measurements and policies |
| Hopkins, L; et al. | 2018 | The process of establishing Discovery College in Melbourne | Mental Health & Social Inclusion | Case study | Australia | Health / education services | A youth-focused recovery college, Melbourne |
| Jeffs, L; et al. | 2019 | Engaging Stakeholders to Co-design an Academic Practice Strategic Plan in an Integrated Health System: The Key Roles of the Nurse Executive and Planning Team | Nursing Administration Quarterly | Case study | Canada | Health services | Integrated care system |
| Larkin | 2015 | On the Brink of Genuinely Collaborative Care: Experienced-Based Co-Design in Mental Health | Qualitative Health Research | Named co-production method | UK | Health services | Emergency inpatient mental health services |
| Lindsay, C; et al. [Lindsay 1] | 2018 | Co-production as a route to employability: Lessons from services with lone parents | Public Administration | Qualitative | UK | Employment services | Employment services for lone parents in Scotland |
| Lindsay, C; et al. [Lindsay 2] | 2018 | Co-production and social innovation in street-level employability services: Lessons from services with lone parents in Scotland | International Social Security Review | Qualitative | UK | Health services | Employment services for lone parents in Scotland |
| Macaulay, B | 2016 | Considering social enterprise involvement in the commissioning of health services in Shetland | Local Economy | Qualitative | UK | Health services | Health services in Shetlands (a remote island group off Scotland, UK) |
| Mader, LB; et al. | 2018 | Inverting the patient involvement paradigm: defining patient led research | Research Involvement and Engagement | Case study | UK | Health services | Polycystic kidney disease |
| Marston, C. et al | 2016 | Community participation for transformative action on women’s, children’s and adolescents’ health | Bulletin of the World Health Organization | Review | Global | Health Services | Women, children and adolescent health |
| McColl-Kennedy, JR; et al. | 2012 | Health Care Customer Value Cocreation Practice Styles | Journal of Service Research | Qualitative | Australia | Health services | city, Adult cancer patients in n=2 private oncology day clinics |
| McGregor, J; et al. | 2014 | The college is so different from anything I have done. A study of the characteristics of Nottingham Recovery College"" | The Journal of Mental Health Training, Education and Practice | Case study | UK | Health / education services | Recovery College, England |
| Mifsud, M; et al. | 2015 | Service appropriation: how do customers make the service their own? | Journal of Service Management | Qualitative | France | Health services | Chronic illness care. |
| Millenson, ML; et al. | 2013 | Turning patient-centeredness from ideal to real: lessons from 2 success stories | Journal of Ambulatory Care Management | Named co-production methods | US | Health services | n=2 case studies of patient-centred care initiatives in healthcare |
| Miller, A et al | 2018 | A Community -Directed Integrated Strongyloides Control Program in Queensland, Australia | Tropical Medicine and Infectious Disease | Case study | Australia | Health services | Disease control, Aboriginal community, Queensland |
| Morton, M. & Paice, E. | 2016 | Co-Production at the Strategic Level: Co-Designing an Integrated Care System with Lay Partners in North West London, England | International Journal of Integrated Care | Case study | UK | Policy | Integrated care policy development in NW London, England |
| Mulvale, G. et al | 2019 | Codesigning health and other public services with vulnerable and disadvantaged populations: Insights from an international collaboration | Health Expectations | Case study | Cross national | Health and social care | Vulnerable groups |
| Murphy, L; et al. | 2015 | A quality improvement initiative in community mental health in the republic of Ireland | Health Science Journal | Qualitative | Ireland | Health services | Community mental health initiative |
| Nicol, E; et al. | 2011 | A co-productive health leadership model to support the liberation of the NHS | Journal of the Royal Society of Medicine | Conceptual | UK | Health services | National Health Service |
| Nies, H | 2014 | Communities as co-producers in integrated care | International Journal of Integrated Care | Conceptual | Netherlands | Health and social care | Care for people with complex needs |
| Nimegeer, A; et al. | 2011 | Addressing the problem of rural community engagement in healthcare service design | Health and Place | Case study | UK | Health services | Health service planning in rural communities in the Scottish Highlands |
| Oertzen | 2018 | Co-creating services - conceptual clarification, forms and outcomes | Journal of Service Management | Review | Cross-national | General public services |  |
| Pestoff, V | 2014 | Hybridity, coproduction, and third sector social services in Europe | American Behavioral Scientist | Conceptual | Europe | General public services | 3rd sector organizations involved in public services |
| Poocharoen, OO; Ting, B | 2015 | Collaboration, Co-Production, Networks: Convergence of theories | Public Management Review | Conceptual | Singapore | General public services | n= 4 case studies of public service delivery (1 in healthcare) |
| Powers, KJ; Thompson, F | 1994 | Managing coprovision: Using expectancy theory to overcome the free-rider problem | Journal of Public Administration Research and Theory | Conceptual | Not stated | General public services | Public services |
| Redwood | 2016 | Integration of research and practice to improve public health and healthcare delivery through a collaborative health integration team model - a qualitative investigation | BMC Health Services Research | Qualitative | UK | Health services | Health Integration Team (HIT) UK |
| Rycroft-Malone | 2016 | Collective action for implementation: a realist evaluation of organisational collaboration in health care | Implementation Science | Case study | UK | Health services | N=3 case studies of Collaborations for Leadership in Applied Health Research  & Care (CLAHRCs), England. |
| Sancino, A | 2016 | The Meta Co-production of Community Outcomes: Towards a Citizens’ Capabilities Approach | Voluntas | Conceptual | Not stated | Health and social care | Civil society, public sector and local government |
| Schaaf | 2017 | From favours to entitlements: a community voice and action and health service quality in Zambia | Health Policy and Planning | Case study | Zambia | Health services | A social accountability programme |
| Seid | 2018 | Co-designing a collaborative chronic care network (C3N) fro inflammatory Bowel Disease: Development of Methods | JMIR Human Factors | Case study | US | Health services | Chronic care |
| Sicilia, M; et al. | 2016 | Public services management and co-production in multi-level governance settings | International Review of Administrative Sciences | Case study | Italy | Health services | Autistic children |
| Sorrentino, M; et al. | 2017 | Health care services and the coproduction puzzle: Filling in the blanks | Administration & Society | Case study | Italy | Health services | Cystic fibrosis (CF) Centre in n=1 Italian hospital. |
| Staniszewska et al | 2018 | Reviewing progress in public involvement in NIHR research: developing and implementing a new vision for the future | BMJ Open | Mixed methods | UK | Health research | Patient and public involvement in health research initiative |
| Sturmberg, JP; et al. | 2010 | Music in the Park. An integrating metaphor for the emerging primary (health) care system | Journal of Evaluation in Clinical Practice | Conceptual | Not stated | Health services | Health systems reform |
| Terp | 2016 | A room for design: Through participatory design young adults with schizophrenia become strong collaborators | International Journal of Mental Health Nursing | Named co-production method | Denmark | Health services | Young adults with schizophrenia |
| Topp | 2018 | The health system accountability impact of prison health committees in Zambia | International Journal for Equity in Health | Case study | Zambia | Health services | Prisoner health |
| Vennik, FD; et al. | 2016 | Co-production in healthcare: rhetoric and practice | International Review of Administrative Sciences | Qualitative | Netherlands | Health services | n=5 Dutch hospitals |
| Walsh et al | 2018 | Towards a New Paradigm of Healthcare: Addressing Challenges to Professional Identities through Community Operational Research | European Journal of Operational Research | Case study | UK | Health services | GP surgery pilot, Scotland |
| Ward, ME; et al. | 2018 | Using Co-Design to Develop a Collective Leadership Intervention for Healthcare Teams to Improve Safety Culture | International Journal of Environmental Research and Public Health | Case study | Ireland | Health services | Healthcare teams working across 11 hospitals, 4 Community Healthcare Organizations and a university |
| Williams, BN; et al. | 2016 | (Co)-Contamination as the Dark Side of Co-Production: Public value failures in co-production processes | Public Management Review | Conceptual | US | Health services | Public health, Boston |
| Wilson, G | 1994 | Co-Production and Self-care: New Approaches to Managing Community Care Services for Older People1 | Social Policy & Administration | Conceptual | UK | Health and social care | Community services for older people |
| Windrum | 2014 | Third sector organizations and the co-production of health innovations | Management Decision | Case study | Europe (4 countries) | Health services | N=4 case studies of innovation networks that include third sector organizations |
| Yokota | 2018 | Lesson Learned from co-design and co-production in a portable health clinic research in Jaipur district, India (2016-2018) | Sustainability | Case study | India | Health services | Portable health clinic, Jaipur |
